# Trust-based health decision-making recruits the neural interoceptive saliency network which relates to temporal trajectories of Hemoglobin A1C in Diabetes Type 1

**DOI:** 10.1007/s11682-023-00816-z

**Published:** 2023-11-14

**Authors:** Helena Jorge, Isabel C. Duarte, Miguel Melo, Ana Paula Relvas, Miguel Castelo-Branco

**Affiliations:** 1 PIDFIF - Inter-University PhD Program in Clinical Psychology, Family Psychology and Family Intervention, Faculty of Psychology and Educational Sciences of Coimbra and Faculty of Psychology of Lisbon, Portugal & Coimbra Institute for Biomedical Imaging and Translational Research, CIBIT/ ICNAS, University of Coimbra, Coimbra, Portugal; 2 Coimbra Institute for Biomedical Imaging and Translational Research, CIBIT/ ICNAS, University of Coimbra, Polo 3, 3000-548 Coimbra, Portugal; 3 Endocrinology, Diabetes and Metabolism Department (SEMD), University and Hospital Center of Coimbra, Coimbra, Portugal; 4 Faculty of Psychology and Educational Sciences & Center for Social Studies, University of Coimbra, Coimbra, Portugal

**Keywords:** Decision-making, Diabetes type 1, Trust, Metabolic control, HbA1C, Dynamics, Neuroeconomics

## Abstract

Experimental approaches in neuroeconomics generally involve monetary utility. Utility in the health domain is relevant in diabetes because constant daily life decisions are critical for self-consequential long-term outcomes. We used fMRI to investigate self-consequent decision-making in the health and economic domains in Type 1 Diabetes Mellitus and controls (*N* = 50). We focused on two critical phases of decision-making: Investment and Feedback (Positive or Negative). Patients showed larger BOLD activation of limbic, and reward/dopaminergic regions in particular in the health trust game. Importantly, the worse the trajectory of metabolic control (increasing HbA1C), the higher the BOLD activity in regions of the interoceptive saliency network. This was manifested by positive correlations between brain activity during investment in anterior cingulate cortex and insula and HbA1c blood level progression. We conclude that the neural correlates of health-consequent decision-making domain involve limbic and reward related dopaminergic regions in Type 1 Diabetes Mellitus. Furthermore, the temporal trajectory of HbA1C blood levels is correlated with neural risk processing in the saliency network. Evidence for differential risk processing in the health versus the neuroeconomic context, and the discovery of a role for the saliency interoceptive network in metabolic control trajectories suggests a new perspective on the development of personalized interventions.

**Supplementary Information:**

The online version contains supplementary material available at 10.1007/s11682-023-00816-z.

## Background

Both economic and health-based decision-making in the real world implicate assessing potential outcome values in the presence of uncertainty in socially complex settings involving other individuals, while processing trust. The theoretical framework behind this type of strategic thinking includes contributions from Game Theory (von Neumann & Morgenstern, 2007), and Theory of Mind (Premack & Woodruff, 1978).

Social decision-making in economic exchanges was early on been studied with one shot Trust Games in a landmark study (Berg et al., 1995). Here, one player, the investor, decides to give an amount of money (all, some, or none – the investment, a measure of trust) to the other player (the trustee), which may be multiplied. Then, the trustee decides which received amount of the money he would like to reciprocate – a measure of trustworthiness. Played as an iterative game, decision-makers can strategically improve their outcomes and develop optimal strategies, adjusting the latter according to the predicted behaviours, beliefs, and intentions of the other players. Camerer and Hare (2013) highlighted four components of making predictions in social decision-making: 1) knowing what other players perceive; 2) knowing how they value observable payoffs; 3) predicting the behaviour of other players either in one-shot game or in the first iteration in a repeated game; and 4) learning how behaviour changes with experience.

Making decisions in social situations requires fast integration of complex information. In the medical context, trust is very often involved in doctor-patient interactions. A review about the neural mechanisms that underlie trust games in the economic context (Bellucci et al., 2017) highlighted that in a multi-round game the trust stage was associated with activity in ventral striatum and that the dorsal striatum was more largely recruited in the feedback stage. In her short review, Tzieropoulos (2013) pointed out that as the repayment of trustee increased the head of caudate nucleus was proportionally more active. Moreover, consistent positive feedback yielded activation in ventral striatum and orbitofrontal cortex, both involved in reward processing (Phan et al., 2010). It can be argued that these regions have a role in reputation formation (building an expectation based on experience which is a learning process, whereby the outcome will activate reward circuitry and feedback evaluation mechanisms Tzieropoulos (2013). When breaking a promise (which instantiates negative feedback) there is increased activity of the anterior cingulate and insular cortices, regions of the saliency network, possibly in relation to conflict monitoring and processing of unfair outcomes. This may trigger psychophysiological responses in particular in non-cooperating trustees (Baumgartner et al., 2009). Moreover, in successive moves (implying learning) the ventral striatum seems to signal reward prediction errors about outcomes and representations of the trustworthiness of the partner (Tzieropoulos (2013). In first moves, the anterior insula is more often activated during decision, which is in line with its role in initial uncertainty of the decision outcome processing. At these stages, the intentions of the others in social exchanges are unpredictable so that trust is always risky (Tzieropoulos (2013).

Concerning Theory of Mind networks, changes in brain activity involve three sets of regions: 1) Superior temporal sulcus, temporal pole and temporoparietal junction, 2) limbic-paralimbic regions and 3) prefrontal cortex. Both mentalizing and empathy influence the valuation–decision system to learn and predict the choices of other players and to guide future behaviour (Chen et al., 2019; Olson & Spelke, 2008; Rilling et al., 2002; Singer & Tusche, 2014; Stallen et al., 2013; Vives & FeldmanHall, 2018).

Trust-based decision-making in the health setting has been barely explored from the neuroscientific point of view, which is a major omission, in particular in which concerns chronic diseases. In lifelong diseases, such as diabetes mellitus, daily risk attitudes can lead to self-consequential long-term outcomes. Doctor-patient social exchanges in the health domain are comparable to economic exchanges in trust games. The level of patient’s engagement following a clinical management decision differs from one individual to another. We speculate that this is intrinsically related to a particular valuation system for health-related actions. This will affect the way decision-making is achieved in the context of interaction with health care providers.

T1DM patients are insulin-dependent, requiring tight monitoring to accomplishing metabolic control, concerning carbohydrate levels and dietary restrictions. Otherwise, they risk hyper/hypoglycemia, ketoacidosis and long-term potential complications as retinopathy, nephropathy, neuropathy, and cardiovascular disease that can lead to extreme and irreversible consequences (American Diabetes Association, 2009).

Here we investigated the neural basis of health-related decision-making in diabetes type 1, a chronic disorder with strong personal impact and its relation to the temporal trajectories of HbA1C (glycated hemoglobin). The rationale is that living with a chronic disease implies a strict habit control which depend on daily decision-making and adaptive behaviour. The neural correlates of such behavioural patterns remain to be unraveled and are relevant because the health domain involves an inherently larger personal conflict. We addressed these questions using functional magnetic resonance imaging (fMRI) in Type 1 Diabetes Mellitus (T1DM) patients and controls to understand the neural mechanisms of trust-based decision-making in the economic and health-related domains. We focused on two phases of decision-making: investment (dependent on trust) and outcome monitoring (comprising either positive and negative feedback). Positive and negative feedback relates to being reciprocated or not and it is calculated by two different delta reward values based on Expected and Feedback values: *Positive Reward* events (to receive more than expected) and *Negative Reward* events (to receive less than expected). We hypothesize that T1DM when compared to controls show differential BOLD activity in brain regions related to conflict monitoring, decision-making and emotion/reward. Importantly, we investigated if these brain regions show activation patterns in relation to Investment and Outcome monitoring which can be associated with temporal trajectories of metabolic control as indexed by HbA1C over multiple time points. Finally, we investigated the neural mechanisms underlying health risk aversiveness for T1DM patients. We hypothesized that risk averse patients (those choosing more often to cooperate with doctors) recruit differentially the brain network related to inhibitory control and goal-directed behaviour, as a function of flexibility and larger self-control as compared to patients with larger health risk-taking, who show a less cooperative profile.

## Methods

### Participants

We recruited 50 adults aged 22–55 years. Twenty-five of them were diagnosed with Type 1 Diabetes (mean age = 38.72, SD = 10.38; age range: 22–55 years, 11 males and 14 females; mean HbA1c = 7.86, SD = 1.29; HbA1c range:5.9–11.6) and the remaining 25 were matched healthy individuals (mean age = 35.08; SD = 8.77; age range: 24–55 years, 10 males and 15 females; HbA1c, 4.98 ± 0.25; HbA1c range, 4.5 to 5.7). Clinical analyses to controls were made at the hospital (*Centro Hospitalar e Universitário de Coimbra*) to assure that no one had diagnosis of diabetes mellitus, diagnosed according to the current World Health Organization criteria. Patients were assessed over an interval of at least 2 years and up to 8 years (with visits every 6 months), allowing to obtain rich dynamic information. HbA1c (measured using ionic exchange high-performance liquid chromatography, Little et al., 2011) trajectories were obtained by retrieving dynamic values over time. A HbA1c trend with progressively increasing HbA1c values may indeed indicate poor metabolic control and this is captured by a slope measure. Frequency of hypoglycemia was measured and we found an association with metabolic control (χ 2 (1) = 7.94, *P* = 0.006, d = 0.62). The subgroup with more impaired metabolic control was more strongly associated with the presence of hypoglycemia. Groups were matched according to gender, age, civil state, and household members. Comparing to healthy participants, there were more patients with stable than instable household income, and patients had lower educational level. There were no differences in cognitive performance (Table 1).

**Table 1:** Demographic characteristics, cognitive results, and self-reported measures in T1DM patients and healthy participants (*N* = 50)

Variables	T1DM (*N* = 25)	Healthy (*N* = 25)	*X*^*2*^	*U*	*gl*	*p*	*d*
*Demographic data*							
Gender (M/F)	11/14	10/15	0.08		1	0.770	0.08
Age (y)	38.72 (10.38)	35.08 (8.77)		240.0		0.159	0.40
Civil state (Single/Couple)	11/14	11/14	0.00		1	1.00	0.00
Household members (1/2/3)^a^	7/14/4	9/15/1	2.08		1	0.353	0.40
Household income B (1/2)^b^	18/7	10/15	5.19		1	0.023	0.60
Residence^c^	13/6/6	25/0/0	15.78		2	< 0.001	
Education level (1/2)^d^	11/14	2/23	8.42		1	0.005	0.90
*Cognitive data*							
Vocabulary	32.28 (3.10)	31.52 (2.41)		256.0	-----	0.261	0.31
Digit memory	14.56 (2.12)	15.88 (3.14)		374.5	-----	0.221	0.34
*RPMT*^e^	8.16 (0.98)	8.12 (0.88)		303.5	-----	0.853	0.05
*Self-report measures*							
Neuroticism	8.16 (4.19)	6.80 (3.50)		269.5		0.403	0.23
Extroversion	11.68 (3.87)	12.12 (4.01)		334.0		0.675	0.11
Impulsivity	54.92 (8.55)	58.40 (6.33)		400.5		0.087	0.49
Inhibitory control	40.68 (7.18)	43.08 (5.58)		382.0		0.176	0.38
Lack of planning	14.81 (4.15)	15.32 (2.76)		335.5		0.654	0.01
Health risk perception	38.56 (9.58)	34.68 (6.51)		250.0		0.224	0.34
Past risk	13.72 (3.82)	15.24 (4.20)		373.5		0.235	0.34
Present risk	12.76 (2.84)	13.44 (4.00)		329.0		0.747	0.09
Delay discounting—context variation	2/23	10/15		7.018		0.008	0.80
Emotional eating behaviour	1.95 (0.83)	2.17 (1.10)		329.5		0.741	0.09
External eating behaviour	2.32 (0.53)	2.78 (0.65)		440.0		0.013	0.75
Restrained eating behaviour	1.94 (0.74)	2.44 (0.91)		420.5		0.036	0.62

^a^Household members (1 = living alone 2 = living as a couple 3 = living with children); ^b^Household income (1 = stable; 2 = unstable); ^c^Residence as distance to health services in spending time (1 = Local city; 2 < 1 h; 3 > 1 h); ^d^Educational level (1 = below 12 years; 2 = above 12 years); ^e^*RPMT* Raven's progressive matrices tests; *BMI* Body mass index

Two patients did not complete all the required fMRI tasks, which were nevertheless also performed out of the scanner. Participants used the response box in the right hand given their handedness. All the subjects had normal or corrected to normal vision. Written consent was obtained from all participants, according to the Ethics Committee of the Faculty of Medicine of the University of Coimbra, guided by Declaration of Helsinki.

### Sub-group analysis within T1DM patients: economic and health risk averse and risk taking profiles

T1DM were also divided according their risk attitude, forming two groups: risk averse and risk taking (RS) for each context. The cut-off point was defined according to the frequency of risky decisions for all participants in all trials. For economic context, risky decision was defined as the “50 euros” selection). For the health context, risky decision was defined as “only 1 prick” (in the economic task, participants invest money and in the health task, number of “pricks” of insulin delivery, a larger number implying a larger health investment; for task details see Fig. 2 and description below). In the economic domain, risk aversiveness was defined (in terms of amount invested) as FREQ (50) ≤ 4 (meaning at least 4 events with a selection equal or below 50); (*N* = 11, mean age = 35.45, SD = 9.02: age range:22–46, 5 males and 6 females) and Risk Seeking as FREQ(50) > 4 (meaning at least 4 events with a selection above 50) (*N* = 14; mean age = 41.29, SD = 10.85, age range:22–55, 6 males and 8 females). Between risk averse and risk taking groups within patients, there were no differences in sociodemographic, cognitive, and clinical features. They differed in disease onset time which is lower for the risk averse group (U = 118.0 *p* < 0.05). In the health domain, risk aversiveness was defined as the frequency of deciding to cooperate more than 1 prick (4 or 6) > 1: [*N* = 14, mean age = 35.07, SD = 10.78: age range:22–53, 7 males and 7 females] and Risk taking as the opposite—≤ 1 prick: [*N* = 11; mean age = 43.36, SD = 8.07, age range:27–55, 4 males and 7 females].

### Risk measures

Risk taking profiles were measured by a comprehensive battery of questions. Personality traits were evaluated by EPQ (Eysenck Personality Questionnaire). Impulsivity was measured using the Behaviour Impulsivity Scale-11 (BIS-11) as risk-related constructs. Additionally, we designed a brief questionnaire where participants were confronted with three types of risk measures (context-dependent Risk, Temporal risk and Delay discounting) to achieve an individual real world risk profile. Considering the intrinsic relation between T1DM and self-control, eating behaviour was also assessed using the Portuguese validated version of the Dutch Eating Behaviour Questionnaire (DEBQ) (van Strien et al., 1986; Viana & Sinde, 2003) evaluating three types of eating styles: restrained (to avoid eating more than was initially defined), external (to eat motivated by external factors such as attractive food smell and appearance) and emotional (to eat in response to emotions).

### Trust games

**Fig. 1:**
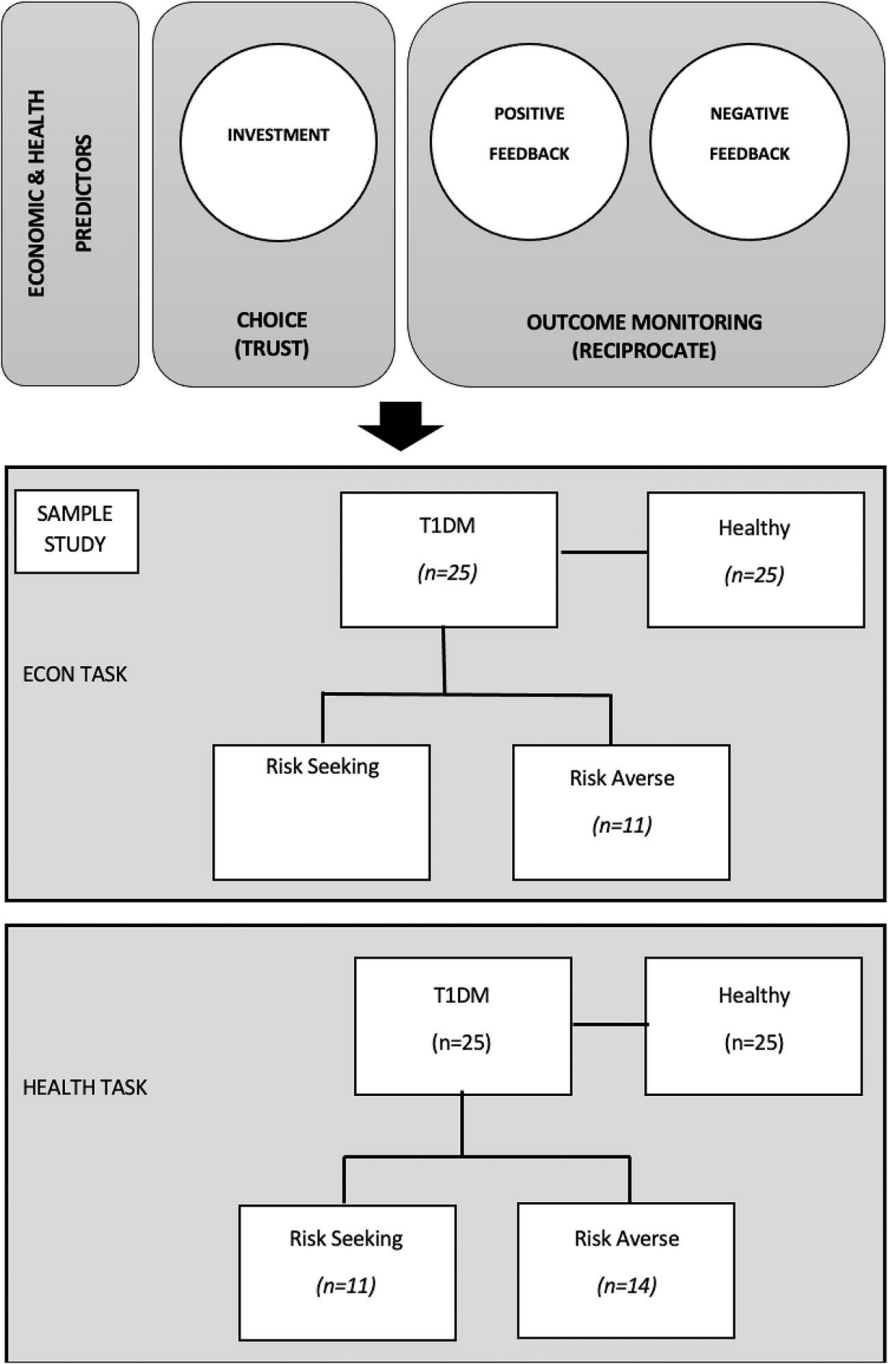
Flowchart of fMRI predictors and study sample

Before the scanning session, the participants were instructed to become familiar with the tasks and with the response box. Participants performed two modified versions of the Trust Game (Berg et al., 1995). We did not triplicate the amount of money as often done (unrealistic for the health game) and the games involved iterative decision-making. Reward outcomes differed according to the context: money in the economic setting and amount of waiting time for consultation as a health-related reward. The scanning sessions consisted of an anatomical run and two functional runs that were counterbalanced to prevent order effects (Fig. 1).

### Scanning session details

In the scanning sessions, for each interaction, participants were presented with a fixation cross for 8 s. The first question (the expected return) is presented in the screen for 8 s (participant time response). A fixation cross was displayed again for 8 s (inter-stimulus interval, ISI). After this period, participants were confronted with the second question (investment or collaboration) for a maximum of 8 s to select their option (leading to a time jitter). After an additional ISI (with fixation cross) of 8 s, the participants were shown the trustee return during 6 s. Both tasks involved iterated interactions with four mediators (trustees) to guide the participant for the best option. Additional details, such as participant instructions, are provided as supplementary material.

#### Analysis of behavioural data

Statistical analyses were conducted in SPSS 24.0. The non-parametric Wilcoxon signed-rank test was used to compare expected value, investment, outcome value and response time between groups (T1DM versus controls) for each context (economic and health). Because *Expected, Investment and Feedback* values have different metrics for both contexts, we transformed data into z-scores. Significance level was considered at *p* < 0.05. The same procedure was repeated to compare other two groups based on task performance (economic and health risk-averse and risk-taking groups).

#### fMRI data acquisition

Structural and functional MRI scans were acquired in a 3 T Magnetom Trio Tim MRI scanner (Siemens, Erlangen, Germany) using a 12-channel head coil. The scanning session included a high resolution T1-weighted MPRAGE sequence that was measured with repetition time (TR) = 2530 ms, echo time (TE) = 3.42 ms, inversion time (TI) = 1100 ms, flip angle of 7º, single shot slices with voxel size 1 × 1 × 1 mm and Field of View (FOV) of 256 mm. Functional images were acquired using BOLD contrast echo planar imaging (EPI), with TR = 2000 ms, TE = 30 ms, voxel size 3 × 3 × 3 mm, and 35 slices covering the entire brain. The task was presented in an LCD monitor (NordicNeuroLab, Bergen, Norway) mounted ~ 156 cm away from the participants’ head. The monitor could be seen through a mirror mounted above the coil. The monitor has a frequency rate of 60 Hz and dimensions of 698.40 × 392.85 mm. The subject could select the response using an MR-compatible response box (Hybridmojo, San Mateo CA, USA) according to three options.

#### fMRI data analysis

Functional images were preprocessed using Brain Voyager (Brain Innovation, Maastricht, the Netherlands) and consisted of slice scan time correction, high temporal filtering, 3D motion correction via realignment, and co-registration to the structural image. Images were transformed into Talairach space for normalization and were then spatially smoothed using a Gaussian kernel of 8 mm of full width at half maximum.

**Fig. 2:**
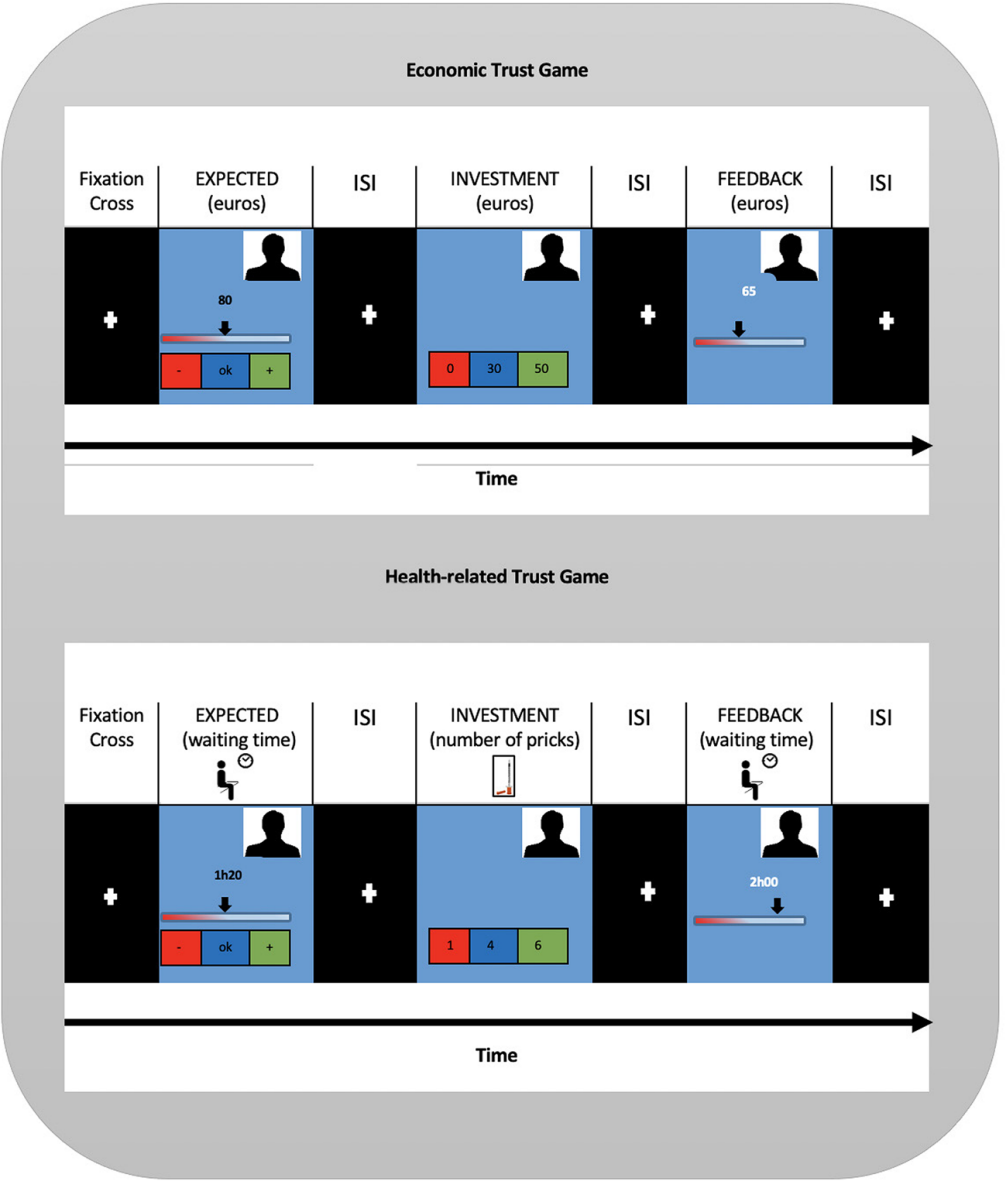
fMRI sequence for economic and health-related trust games. In economic trust game, participants invest money (0, 30, 50 euros) whereas in health trust game, number of pricks (1, 4 or 6). 30 euros means optimal choice and 6 pricks high collaboration. *Positive and Negative Feedback* predictors were obtained by calculation of the difference between Expected and Feedback values for each iteration

We defined three predictors: *Investment, Positive Feedback and Negative Feedback*. Investment was defined as the moment participants had to choose one of the three risk options (0, 30, 50 euros or 6, 4, 1 pricks, depending on the experimental task, see Fig. 2). *Positive and Negative Feedback* predictors were obtained by calculation of the difference between expected and feedback values for each iteration. Groups analysis were performed to compare T1DM patients versus Controls or to compare Risk-Averse versus Risk-Taking subgroups. To correct for multiple comparisons a cluster-level thresholding method was used at a fixed p-value of 0.05. The method estimates, using Monte Carlo simulations (1000 iterations), the minimum cluster size for each map. The number of contiguous voxels used as the threshold extension is presented in the results section for each map. Finally, for each individual we estimated a line that better fits the individual values of HbA1c over time (see Supplementary Fig. 1). The slope of that line indicates whether each participant's trend is to increase or decrease the HbA1c value. Please note that computing the slopes as trendlines over multiple time points may result in slopes that do not intersect every point in some cases. The slope of each patient was used to define successful metabolic control (negative slope, i.e. decreasing HbA1c values over time) and impaired metabolic control (positive slope, i.e. increasing HbA1c values over time).

##### Data and resource availability

Data and resources are available upon reasonable request to the corresponding author.

## Results

### Behavioural risk measures

#### T1DM and healthy groups

*Questionnaire based Self-reported measures of risk-taking—*Mann–Whitney tests revealed that T1DM patients and controls do differ in choice measures of delay discounting. Controls tend to opt for (larger) delayed rewards in all contexts (stable choice), the T1DM patients chose delayed rewards only in the diabetic health domain (Table 1). Regarding eating behaviour, people with T1DM reported lower scores in external and restrained eating behaviour scores (neither eating longer based on food attractiveness nor avoiding eating less) as compared to the healthy control group.

Concerning Trust Games behavioural results acquired during the fMRI experiment (Table 2), non-parametric independent sample tests revealed significant differences between groups for clinical expected value, because T1DM patients expected in general larger waiting times than they received as compared to control participants.

**Table 2:** Behavioural results in economic and health trust games

A												
	*Economic Trust Game*											
	T1DM (*N* = 25)					Controls (*N* = 25)						
Variables	M	SD	1stQ	2ndQ	3rdQ	M	SD	1stQ	2ndQ	3rdQ	*U*	*p*
Investment	27.52	8.85	17.14	28.57	32.85	26.35	8.49	25.00	30.72	32.14	286.5	0.614
Expected	72.86	10.68	66.34	73.57	81.25	72.08	9.2	69.05	74.71	77.45	322.0	0.854
Feedback	85.81	14.36	74.11	89.64	96.25	88.80	15.82	84.11	94.46	97.50	244.0	0.184
*Health trust game*												
Variables	M	SD	1stQ	2ndQ	3rdQ	M	SD	1stQ	2ndQ	3rdQ	*U*	*p*
Investment	4.62	1.11	3.93	4.78	5.57	4.85	1.11	4.55	5.22	5.57	258.5	0.294
Expected	113.79	22.44	98.39	112.50	136.07	100.1	24.33	89.46	98.93	117.50	415.5	**0.046**
Feedback	128.29	20.22	113.57	128.93	141.43	119.3	16.81	107.14	113.93	130.36	395.0	0.107
B												
*Economic trust game*												
	Risk averse *N* = 11					Risk seeking *N* = 14						
Variables	M	SD	1stQ	2ndQ	3rdQ	M	SD	1stQ	2ndQ	3rdQ	*U*	*p*
Investment	22.46	8.09	15.00	23.58	30.00	31.36	7.31	25.00	32.86	36.43	127.5	**0.004**
Expected	73.45	11.05	65.94	74.02	81.79	72.15	10.66	65.18	73.22	80.00	69	0.687
Feedback	83.71	15.96	70.72	83.13	95.54	88.48	12.22	81.78	92.86	96.25	91.0	0.467
*Health trust game*												
	Risk averse *N* = 10					Risk seeking *N* = 15						
Variables	M	SD	1stQ	2ndQ	3rdQ	M	SD	1stQ	2ndQ	3rdQ	*U*	*p*
Investment	5.56	0.41	5.15	5.71	5.95	3.98	0.98	3.86	3.96	4.72	200.0	** < 0.001**
Expected	113.57	21.90	101.78	112.32	134.29	113.9	23.55	93.22	112.50	136.79	75.0	1.000
Feedback	116.07	23.23	99.64	110.71	124.38	136.4	13.28	128.93	133.93	144.29	130.0	** < 0.001**

Between groups analysis for T1DM and healthy groups (*N* = 50) showing that they are largely matched considering Investment, Expected Value and Feedback (A). Significant differences in particular considering Investment and Feedback (Health game) for Risk-Averse and Risk-Taking between groups analysis within patients with T1DM (*N* = 25) considering Investment, Expected Value and Feedback (B)

Significant differences, *p* < 0.05, are marked in bold

#### Risk-averse and risk-taking groups (within group T1DM analysis)

Concerning Self-reported measures Mann–Whitney tests revealed no significant differences between risk averse and risk taking T1DM profiles in neuroeconomic and health contexts.

Regarding Trust Games Non-parametric independent sample tests revealed significant differences between groups for investment in both contexts (see Table 2). Additionally, in the health setting, risk taking patients (less collaborative) receive more waiting time than risk averse patients (worse feedback in the health trust game).

### Neuroimaging: T1DM recruit into larger extent reward and emotional processing regions in the health task 

#### T1DM and controls

##### Investment in the economic trust game

**Fig. 3:**
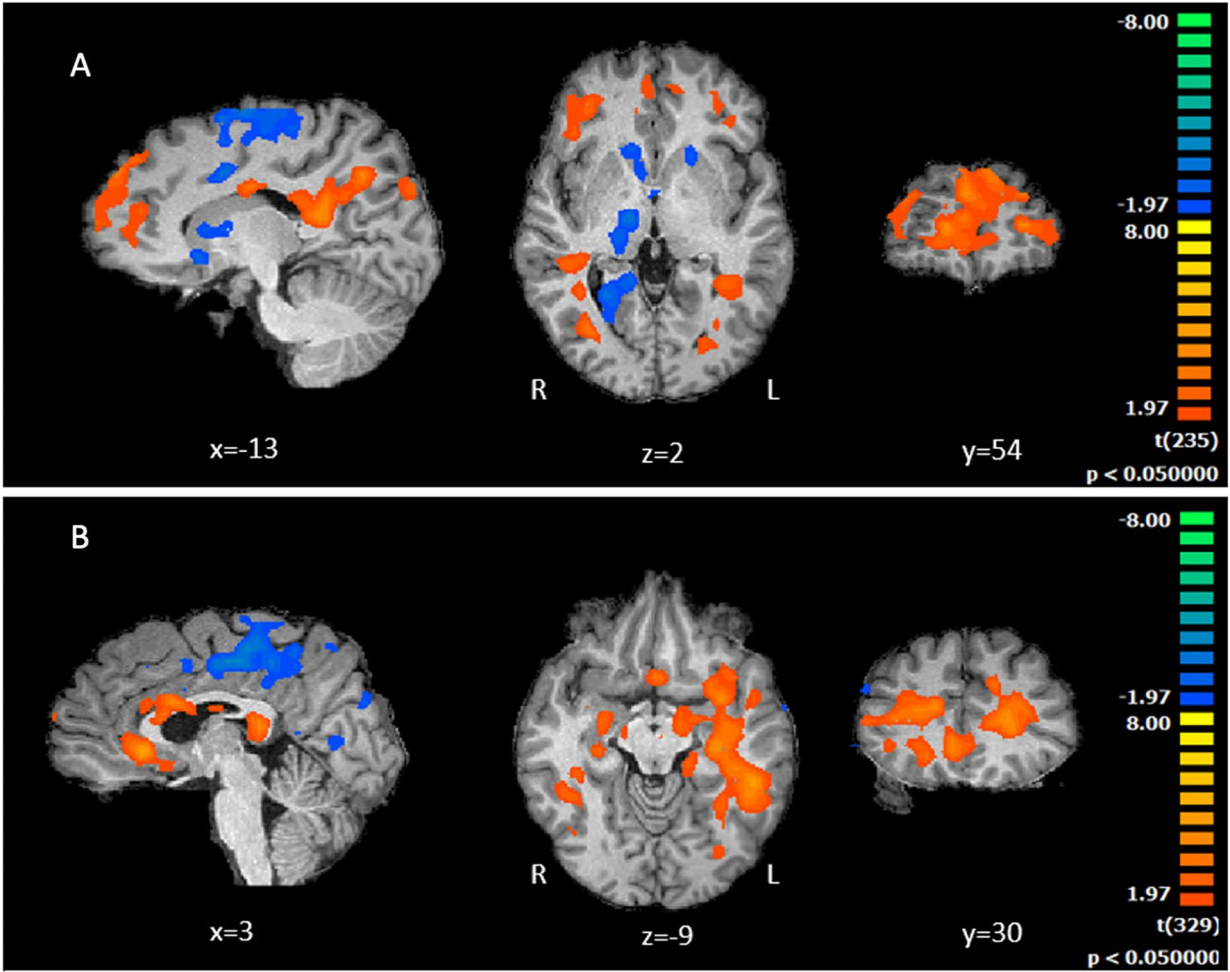
A fMRI whole brain comparison between the T1DM and Control Groups during economic and health investment conditions. **A** Statistical maps for the comparison between T1DM and Controls during the economic investment condition. Concerning the contrast (T1DM > Controls) included posterior cingulate cortex (BA23,30,31) and middle frontal gyrus (BA9, BA10). Controls activated into a larger extent the parietal cortex (BA39, BA40), and in particular bilateral anterior caudate, (minimum cluster size 107). **B** Statistical maps for the comparison between T1DM and Controls during health investment condition. T1DM patients showed larger limbic activation (subgenual cingulate cortex (BA25) and amygdala), as well as memory regions (hippocampus and parahippocampus) and prefrontal regions [medial (BA10, BA46) and inferior (BA45, BA47)]. Controls recruited again parietal regions (BA39/BA40) (minimum cluster size 108)

We carried out a whole-brain group comparison between T1DM and controls while they performed the investment condition (Fig. 3, A). Patients activate into a larger extent medial and anterolateral prefrontal cortical areas, posterior cingulate regions and control participants the head of the caudate nucleus and parietal cortex.

##### Investment in health-related trust game

Similar analysis was carried out comparing T1DM patients with healthy participants during health related investments (measured by the number of accepted insulin pricks, Fig. 3B). Patients differ from controls in limbic regions (subgenual ACC (BA25) and amygdala), dopaminergic midbrain reward regions, memory regions (hippocampus and parahippocampus) and prefrontal regions [medial PFC, dorsolateral PFC (BA10, BA46) as well as regions involved in inhibitory control such as the inferior frontal gyrus (BA45, BA47)]. Conversely, controls showed larger activation outside the limbic system and in particular again larger higher activity in the caudate while patients showed higher activity in the putamen (as well as midbrain regions), suggesting that the controls are more goal oriented as compared to patients.

##### Positive and negative feedback

For the *Positive Reward predictor,* no differences were found when comparing T1DM with the control group, in both contexts. In contrast, *for the Negative Reward predictor, in the health setting* (receiving more waiting time than expected) the T1*DM versus control group contrast* showed larger BOLD response in bilateral hippocampus and right parahippocampus.

#### Risk-averse and risk-taking T1DM groups

##### Investment in economic trust game

**Fig. 4:**
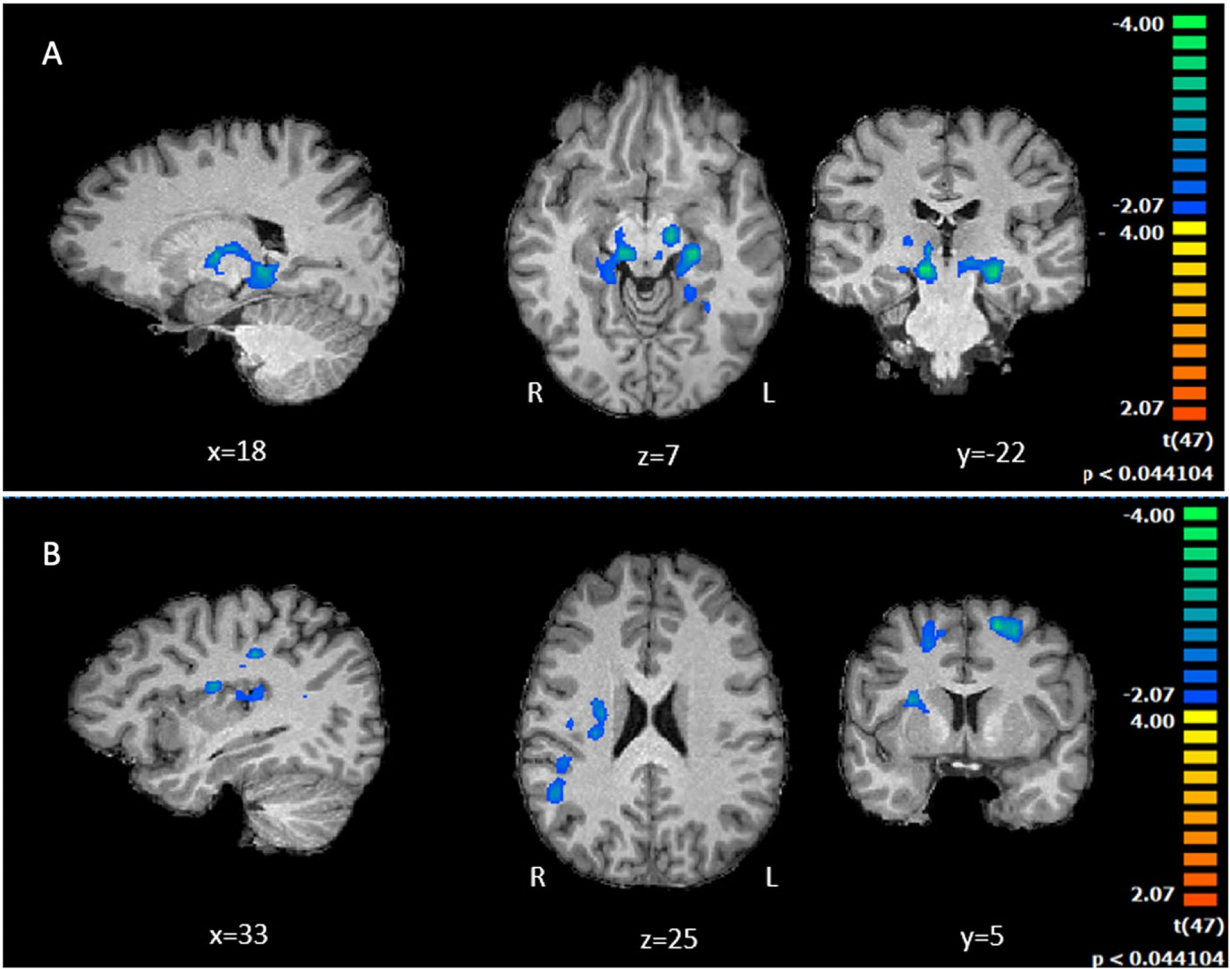
A fMRI Whole Brain activation between patients for risk averse and risk taking profiles contrasts for economic (**A**) and health investment (**B**). **A** Differential Brain activations were found in thalamus, dopaminergic regions (substancia nigra, ventral tegmental area), memory (hippocampus, parahippocampus) and limbic (amygdala) for the risk seeking group (investing more money) (minimum cluster size 38). **B** Statistical maps for the comparison between Risk Averse and Risk Seeking T1DM patients during the Health Investment condition. Brain activation within patients with less health collaboration, (risk taking patients, whose options for no collaboration with doctors were more frequent), showed larger activations in parietal (BA39, BA40) and posterior insular cortex (minimum cluster size 33). Note the absence of positive cluster (no larger activation for risk taking profiles)

Comparing risk-averse versus risk-taking patients during the economic investment, we found higher activity from the risk-taking individuals in a set subcortical structures involved in dopaminergic reward and emotion processing: thalamus, the ventral tegmental area (VTA), substantia nigra, hippocampus, parahippocampus and amygdala (Fig. 4A).

##### Investment in the health trust game task

When comparing risk-averse versus risk-taking patients, in which concerns health-related investment, we found increased larger BOLD responses in risk-taking patients, whose options for no collaboration were more frequent, in parietal (BA40, BA39) temporal regions (BA21), putamen and posterior insular cortex, showing larger recruitment of decision-making and interoceptive processing regions (Fig. 4B). No significant differences were found between risk-averse and risk-taking patients for positive and negative rewards.

#### Brain regions associated with evolution of metabolic control (HbA1C) profiles

##### Investment in both contexts—a role for the saliency network

**Fig. 5:**
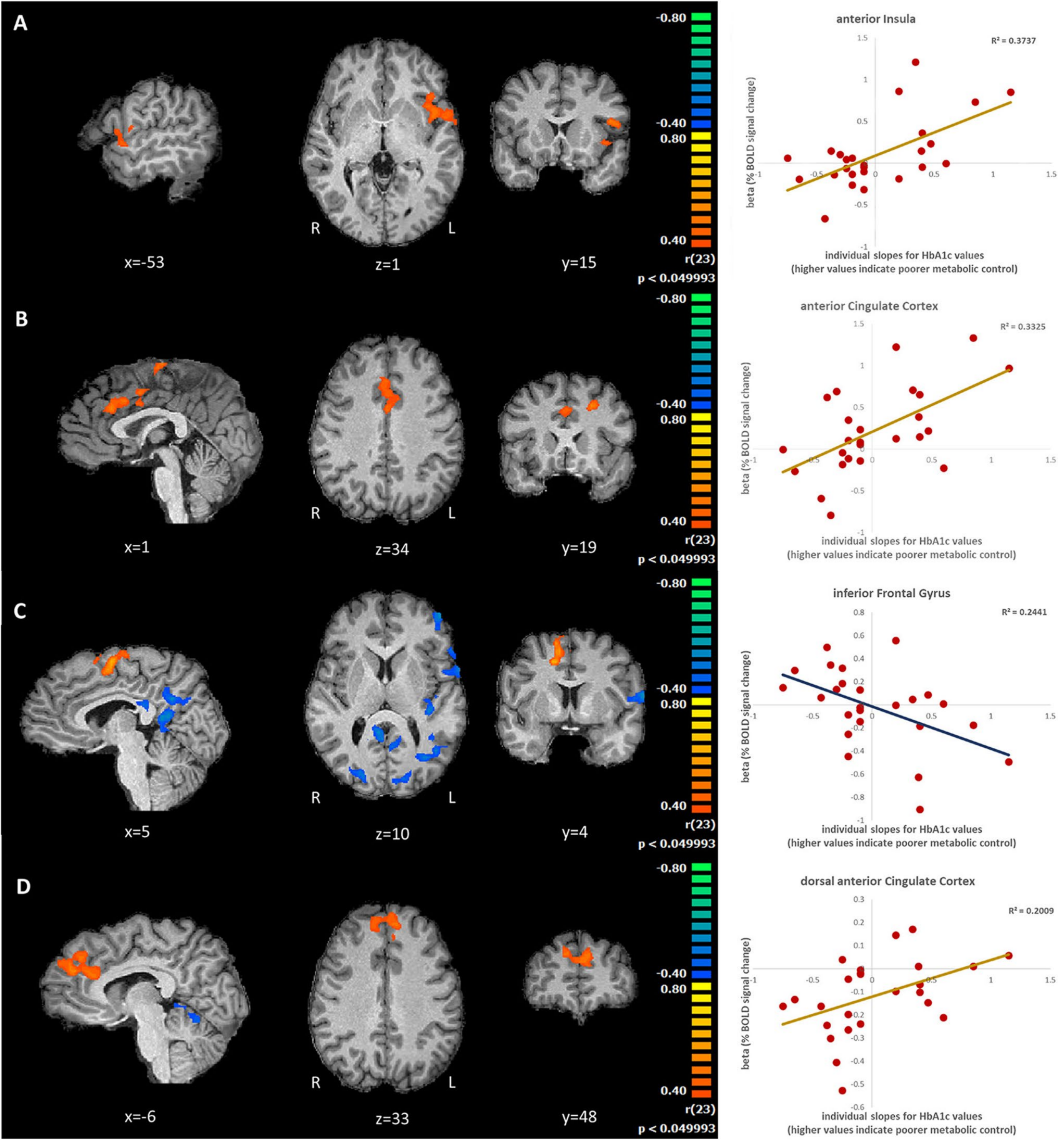
A fMRI Whole brain correlation analysis between evolution of HbA1c trajectories and the BOLD activity, and a correlation plot of ROIs, during the economic investment (**A**), health investment (**B**), negative reward in economic trust game (**C**) and positive reward in health trust game (**D**) performed by the T1DM patients. **A** Economic Investment condition and HbA1c (higher evolving HbA1c values reflecting a poorer metabolic control). A positive value (red) for the correlation, means that poorer the metabolic control (higher the evolution of HbA1c), higher the BOLD activity during the Economic Investment condition. Note the anterior insula modulation within the salience network (minimum cluster size 76). **B** Health Investment condition. Note dorsal ACC activation within the saliency network (minimum cluster size 100). **C** Whole brain correlation analysis between BOLD activity during Negative Reward condition (Receiving less money than expected) under the economic Investment condition. Note that successful metabolic control (blue) is associated with correlated activations in posterior cingulate, inferior frontal gyrus, middle temporal gyrus and posterior insula. (minimum cluster size 76). **D** Whole brain correlation analysis between BOLD activity during Positive Reward in the health trust game (receiving less waiting time than expected) condition and HbA1c (higher HbA1c values reflecting a poorer metabolic control). Brain activity correlated with impaired metabolic control in bilateral ACC within the saliency network and middle frontal gyrus (BA9) activation (positive correlations-red) (minimum cluster size 99). Note the ROI betas values extracted to perform the correlation plots were selected from the respective correlation maps after application of threshold at the significance level

Correlations maps were calculated between the BOLD activity during choice condition and the metabolic control profile as given by the trajectories (slopes) of HbA1c value over time **(**see methods and Supplementary Fig. 1) (higher values of HbA1 evolution meaning poor metabolic control). In the economic task, correlation between neural activity and variation of HbA1c revealed a striking positive correlation with activity in a region of the saliency network, the anterior insula (Fig. 5A). Associations were also found with middle frontal gyrus (BA9, BA10), the inferior frontal gyrus (related to impulsivity). In sum, the correlation between brain activity and impaired metabolic control dominates in the insulo-opercular complex, a hub within the saliency network (see scatterplots in Fig. 5). The poorer the metabolic control, the higher the BOLD activity in these areas, related to decision. In the health task, there was a positive correlation between metabolic trajectories (increased values of HbA1c meaning poorer metabolic control) and BOLD response in Anterior Cingulate Cortex (BA24 and BA32)- the anterior hub of the saliency network (Fig. 5B). In sum two key regions of the saliency network are related to metabolic control.

##### Relation between metabolic control trajectories and brain activity in the context of positive and negative rewards

In the economic setting (Fig. 5C), in the context of responses to negative rewards (receiving less money than expected) we found restricted patterns of activation in association with impaired metabolic control (correlation between neural activity and variation of HbA1c). In contrast, better metabolic control had an association with BOLD responses in the posterior left insula, inferior frontal gyrus (BA44), superior parietal lobe (BA7) and posterior cingulate cortex (BA21,23).

In the health setting (Fig. 5D), and the context of positive rewards (receiving less waiting time), we found a positive correlation (increasing HbA1c meaning poor metabolic control) with activity in dorsal ACC (the anterior hub of the saliency network) as well as prefrontal regions (BA9 and BA8).

## Discussion

To the best of our knowledge, this is the first fMRI study that examined the neural correlates for trust-based decision-making in the economic and health context, with a link with metabolic trajectories, in a chronic condition with lifelong disability, diabetes mellitus. This follows up two prior behavioural studies from our group suggesting behavioural partitions between risk averse and risk taking patients (Jorge et al., 2021, 2022b). In another prior study of the neural correlates of cognitive impulsivity in T1DM, we found group differences in the interoceptive saliency network (Jorge et al., 2022a), which we show here to be also present under trust based decision making conditions, in particular in the health related doctor-patient contexts. We were interested in understanding how decision-making under uncertainty relates with the temporal trajectory of HbA1c (as measured by the respective trend line). Our findings can be summarized as follows: 1) overall, T1DM patients differ from controls particularly in choice (investment). During outcome monitoring, neural responses to the latter were tightly linked to metabolic control in patients. 2) Our findings indeed indicated that there was a significant association between neural activity and impaired metabolic control, highlighting the role of HbA1c in risk processing. 3) we also gathered evidence for activation of reward, emotion and in particular the salience processing network (anterior cingulate and insula, which relate to interoceptive processing) in patients. 4) Concerning the role of context, our findings suggest that the health context is deeply self-consequential with high impact in emotional, memory and rewards circuits, in patients.

Controls showed in general larger BOLD responses in subcortical caudate regions, suggesting a larger involvement of goal oriented processes. Activation of the putamen was in contrast observed in patients, in particular the risk taking ones, which likely related to a more repetitive habitual action style, thereby rendering them less sensitive to trial and error learning rules.

Patients were less sensitive to positive feedback (less waiting time) possibly given their prior experiences in health care services, which trigger autobiographic memories, as suggested by activation in memory circuits. Bilateral activations in the hippocampus and parahippocampus for negative feedback (waiting more time than expected for consultation) indeed suggested an enhanced role for memory mechanisms for patients. This is consistent with behavioural results in terms of expected value. This might also explain why patients maybe processing aversive stimuli (pricks and waiting time) in a way that activates the putamen in relation with implementation of habitual actions triggered by increased anxious states (Banca et al., 2015).

Patients that were risk-taking in the economic trust game showed activation in brain areas related to the limbic system and dopaminergic midbrain regions related to motivation and reward (Ilango et al., 2014), such as the ventral tegmental area. For the health context, patients with absent collaboration profiles (Risk-taking) showed activations in parietal (BA40, BA39) and temporal (BA21) regions, putamen, and posterior insula related to interoceptive processing.

### Correlation between neural activity and variations of HbA1c

When assessing correlations for trajectories of metabolic control, as measured by HbA1C across multiple time points, a role for the saliency network emerged again (anterior insula and anterior cingulate cortex in economic and health investment) in particular in association with impaired metabolic control. Successful control was related to activation of posterior cingulate, posterior insula and parietal cortex. In general, the effect of biological worsening (positive slope of HbA1c), showed an association with regions related to inhibitory control, error monitoring/conflict and interoceptive processing, with a pivotal role for the saliency network.

In relation to positive and negative feedback, in the health setting, impaired metabolic control is associated with responses also mainly in regions of the saliency network (in particular ACC) as well as MFG. These regions are also related to emotional processing (Etkin et al., 2011). In the economic setting, successful metabolic control is associated with activation in brain regions related to signalling the aversive interoceptive outcomes related to “unfair offers” (Clark et al., 2008; Krueger et al., 2020) such as left posterior insula or impulse control (the Inferior Frontal gyrus).

In sum, for investment, positive or negative feedback, biological changes in the health context were related to saliency, impulse control and emotional processing brain areas.

The main focus of this study was to understand the neural basis of trust-based decision-making in the health domain, in particular in which concerns associations with trajectories of metabolic control.

Some limitations have to be acknowledged, in particular regarding sample size for secondary analysis questions regarding the role of different types of trust and health game mediators, providing different reward and punishment ranges of values.

To sum up, we have found that impaired trajectories of HbA1c and metabolic control are associated with increased BOLD responses in saliency network regions (anterior cingulate and insula) in a chronic lifelong disorder, Type 1 Diabetes. Health contexts were emotionally more relevant and required hard self-consequent decision for patients and lead to stronger responses in reward, emotion and memory related regions, in the health setting. This study represents a novel approach bridging neuroeconomics and “healthconomics”, translating to the health context the neural correlates of human trust-based decision-making, based on biological and neuropsychological features within and between clinical and healthy populations.

### Supplementary Information

Below is the link to the electronic supplementary material.

Supplementary file1 (PDF 1.01 MB)

## Data Availability

The datasets generated during and/or analyzed during the current study are not publicly available due to the fact that they contain private personal and medical information but are available from the corresponding author on reasonable request.
