# Supplementary material for: Trust-based health decision-making recruits the neural interoceptive saliency network which relates to temporal trajectories of Hemoglobin A1C in Diabetes Type 1
Source: Brain Imaging Behav. 2023 Nov 14;18(1):171–83. doi: 10.1007/s11682-023-00816-z (PMC10844148; doi:10.1007/s11682-023-00816-z)
Supplement: Supplementary file 1 — Supplementary file1 (PDF 1.01 MB) [file 11682_2023_816_MOESM1_ESM.pdf]

Supplementary material

Supplementary Figure 1

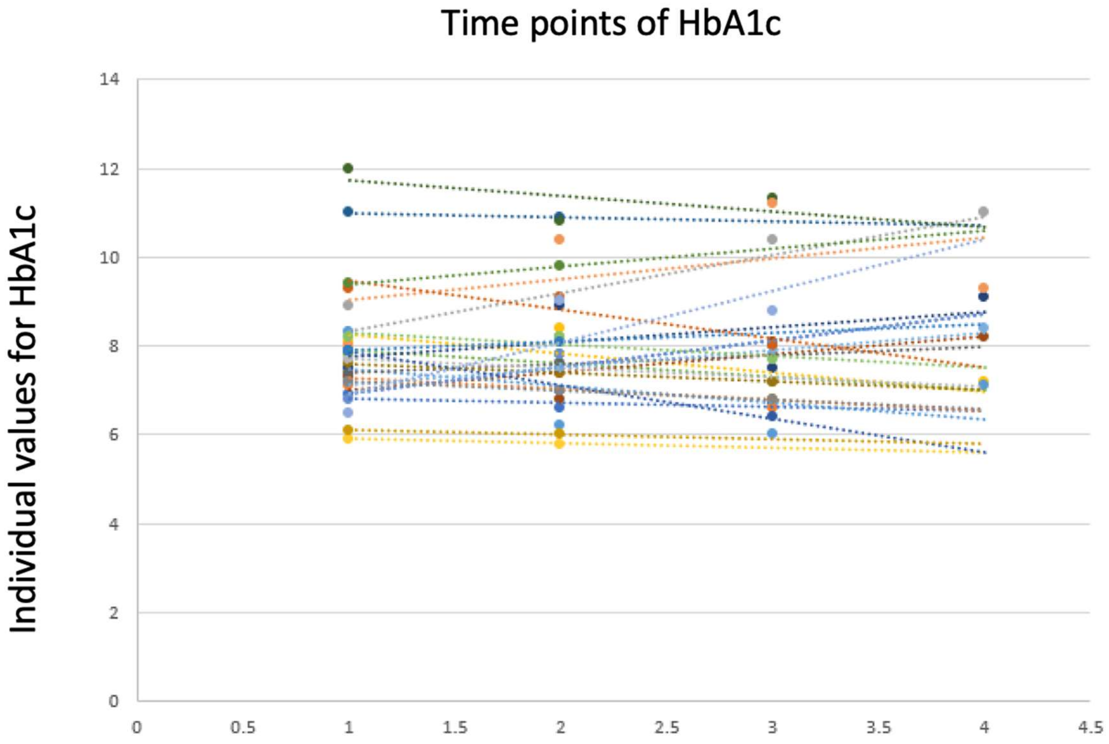

Supplementary Figure 1. Individual trajectories of HbA1C across time

Table S1. Differences in BOLD responses. Differences in activation T1DM and Controls between group analysis for economic (A)and health investment(B). Correlation between neural activation and variations of metabolic control within T1DM patients (C) Between group analysis for Risk averse and risk seeking groups within patients in economic and health investment (D)

Anatomical Region	BA	H	*Talairach* (peak voxels)			T-max	P<
**A. T1DM and control contrasts for economic investment**							
**Economic investment T1DM>Controls**							
Posterior cingulate gyrus	30,31	L	-15	43	16	4.98	0.000001
	23	L					
Middle frontal gyrus	9,10	L	27	11	31	4.99	0.000001
**Economic Investment Controls>T1DM**							
Anterior cingulate gyrus,	24	R	27	-10	55	-5.82	0.00000
Posterior cingulate gyrus,	31	R					
Inferior parietal lobe	39	R					
	40	R					
Caudate, putamen, globus pallidus,thalamus	48,49,51,50	R	6	-13	-8	-4.43	0.000014
Fusiform, visual associatiom and	37,19	R	21	-64	7	-4.36	0.000019
posterior cingulate gyrus	23						
Caudate, putamen, insula	48,49,13	L	-18	14	10	-4.37	0.000018
Lateral premotor area, and	6, 8	R	9	11	43	3.51	0.001
anterior cingulate gyrus	24	R					
posterior cingulate gyrus	23	R					
Middle frontal gyrus	9,10	R	1	50	37	3.02	0.004

Anatomical Region	BA	H	*Talairach* (peak voxels)			T-max	P<
**B. T1DM and Controls contrasts for health investment**							
**Health Investment T1DM>Controls**							
Superior Sensoriomotor Cortex	7	L	-24	-52	31	7.45	0.000000
PMA	6	L	-6	14	64	3.63	0.00031
Middle frontal Gyrus	10	B	-42	54	13	4.23	0.00030
aMFG	46	R					
Inferior Frontal Gyris-pars Triangularis	45	B					
Inferior Frontal Gyrus –Pars orbitalis	47						
Parahippocampus	36	L	-3	-37	1	3.36	0.00086
Hippocampus	54	B					
Amygdala	53	B					
Insula	13	R					
Putamen	49	B					
Thalamus	50	B					
Superior Temporal Gyrus	22	R					
Middle Temporal Gyrus	21	R					
Temporal Lobe	38	L					
Sugenual Gyrus	25	L					
Anterior cingulate cortex	32	L					
Posterior cingulate cortex	23	L					
**Health Investment Controls>T1DM**							
Middle Temporal Gyrus	21	R	61	-19	-11	-3.03	0.002
Superior Sensoriomotor Cortex	7	L	-24	-52	31	7.45	<0.001
IPLobe_Supramarginal Gyrus	40	R	57	-19	13	-4.59	<0.001
IPLobe_Angular Gyrus	39	R	63	-43	23	-4.75	<0.001
Superior Temporal Gyrus	22	R	60	-4	4	-3.82	0.0001
PMA	6	R	57	-7	43	-4.27	0.001
IPLobe_Angular Gyrus	39	R	45	-67	28	-4.75	<0.001
Middle Frontal Gyrus	10	R	39	59	7	-4.43	0.000013
Insula	13	R	30	-25	13	-3.88	0.0009
Visual	19	R	12	-76	34	-3.95	0.000096
PMA	6	R	9	-7	67	-4.26	0.000026
Fusiform	37	R	24	-52	-8	-3.56	<0.0001
PFC-PMA	8	R	3	38	46	-3.22	0.001
Anterior Cingulate Cortex	32,24	L	-9	14	37	-5.13	<0.001
Posterior Cingulate Cortex	23,31						
Caudate ,Thalamus	48	L	-9	-4	13	-3.63	0.0004
Superior Sensoriomotor Cortex	7	L	-18	-67	49	-3.24	0.001
PFC-PMA	8	L	-27	32	43	-2.62	0.0009
IPLobe_Angular Gyrus	39	L	-33	-70	47	-2.92	0.003
PMA	6	L	-39	-7	46	-3.73	0.000231
Fusiform	37	L	-63	-53	1	-3.47	0.0005
IPLobe_Angular Gyrus	39	L	-60	-40	38	-3.34	0.00093
IPLobe_Supramarginal Gyrus	40						
Thalamus, Putamen,Globus Pallidus, Insula	50,49,51,13	L	4	65	7	4.32	0.0003

Anatomical Region	BA	H	*Talairach* (peak voxels)			T-max	P<
**C. Correlation with variation of HbA1c within patients**							
**Economic Investment Impaired metabolic control (red)**							
Middle Frontal Gyrus, Inferior Frontal Gyrus	10,9,44	L	-42	20	22	0.63	0.000605
Insula	13	L					
**Health Investment Impaired metabolic control (red)**							
Anterior Cingulate Cortex	32,24	L	-21	17	40	0.60	0.0013
**Positive Reward Impaired metabolic control Health Context**							
Anterior Cingulate Cortex	32	L	-12	14	37	0.55	0.003
SMA	8	L					
Middle frontal gyrus	9	L					
Anterior Cingulate Cortex	32	R	12	32	16	0.57	0.002
**Negative Reward Correlation with variation of HbA1c Impaired metabolic control Economic Context**							
Lateral premotor area	6	R	6	-4	58	0.73	0.000031
**Negative Reward Correlation with variation of HbA1c Sucessful metabolic control Economic Context**							
Posterior cingulate Cortex	23	R	6	-46	10	-0.58	0.002
Superior Parietal Lobe	7	R	21	-61	34	-0.72	0.000048
Inferior Frontal Gyrus	44	L	-61	8	13	-0.59	0.00017
Middle Temporal Gyrus	21	L	-67	-16	-2	-0.67	0.0000
Posterior insula	13	L	-31	20	7	-0.41	0.036

Anatomical Region	BA	H	*Talairach* (peak voxels)			T-max	P<
**D. Risk averse and risk seeking profiles within patients**							
**Economic investment Risk seeking>risk averse**							
Thalamus,	50	R	12	-22	5	-3.28	0.00058
Hipocampus,	54	R	20	-31	-2	-2.892	0.008
parahippocampus,	36	L	-19	-29	-5	-2.75	0.006
amygdala	53	L	-14	-4	-11	-2.4	0.02
**Health Investment Risk seeking>Risk averse**							
Inferior parietal lobe	39	R	40	-54	17	-2.47	0.021
IPL_supramarginal Gyrus	40	R	33	-28	37	-2.96	0.006
Lateral premotor area	6	L	-19	12	59	-2.59	0.006
Middle temporal lobe	21	R	48	-38	7	-2.37	0.02
Insula	13	R	35	-26	12	-2.28	0.03
Putamen	49	R	23	-2	12	-1.26	0.02

Experimental instructions and design details

♦ *Instructions for economic and health trust games*

You will play a game with 4 mediators for 7 rounds. In each round they will appear at random way. You will recognize them through the face image or the image of a computer (once one of them is a computer) as you can see in this example [in the instruction, we showed only a silhouette of a human face to the participant]. What will happen then? On every move with a trustee, you have to answer to two questions. First question: How much money do you expect to receive? It can range from 40 to 240 euros, pressing the buttons to the left or to the right to find your final option (pressing ok, the middle button). Second question: How much do you want to invest? Here, you will be confronted with three options: O, 30 or 50 Euros. The order of the buttons corresponds to the order of the option presentation (blue, red and green). After your selection, you will be presented with the trustee return, that can be more or can be less than what you initially expected. So, the next time you play with this specific player you can decide if you want to keep your investment or change it. It is especially important to pay attention to each player's return. What remains to be said? Each player has a different way of return so throughout the game you will discover the best option of investment with each one. The main goal of the game is to earn money. I can say that 0 option gives you a small and fixed return and only the 50 option can lead to a jackpot return. Do you have any doubt?[…]

Ok, I will ask you to play another game that has exactly the same structure but instead of economists you will play with doctors from a fictitious endocrinology service which has the following rule: if you decide to collaborate for a successful treatment you spend less time waiting for consultation. *On every move with one of three doctors or a computer, you have to answer to the* first question: How much

time do you expect to wait for consultation? After that, they will ask you to choose between 1, 4 or 6 pricks that means how much do you want to collaborate for a successful treatment: a little bit(1), a little (4) or a lot(6). In exchange, they will offer more waiting time for consultation or less time, according to two reasons: your commitment option and the doctor profile. The fact that there are rules does not mean that they are followed. The main goal of this game is to wait as little time as possible for the consultation. So, play attention to your options and the doctors return to decide if you would like to change or not your commitment next time you play with this trustee. *Do you have any doubt? [to participants who belonged to the healthy group, we made a short introduction to diabetes disease so that they could understand the relation between pricks and successful treatment]*

- ♦ *Scanning session details*

In the scanning sessions, for each interaction, participants were presented with a fixation cross for 8s. The first question (the expected return) is presented in the screen for 8 s (participant time response). A fixation cross was displayed again for 8 s (inter-stimulus interval, ISI). After this period, participants were confronted with the second question (investment or collaboration) for a maximum of 8s to select their option (leading to a time jitter). After an additional ISI (with fixation cross) of 8s, the participants were shown the trustee return during 6s.

♦ *Economic Trust Game and payoff contingencies*

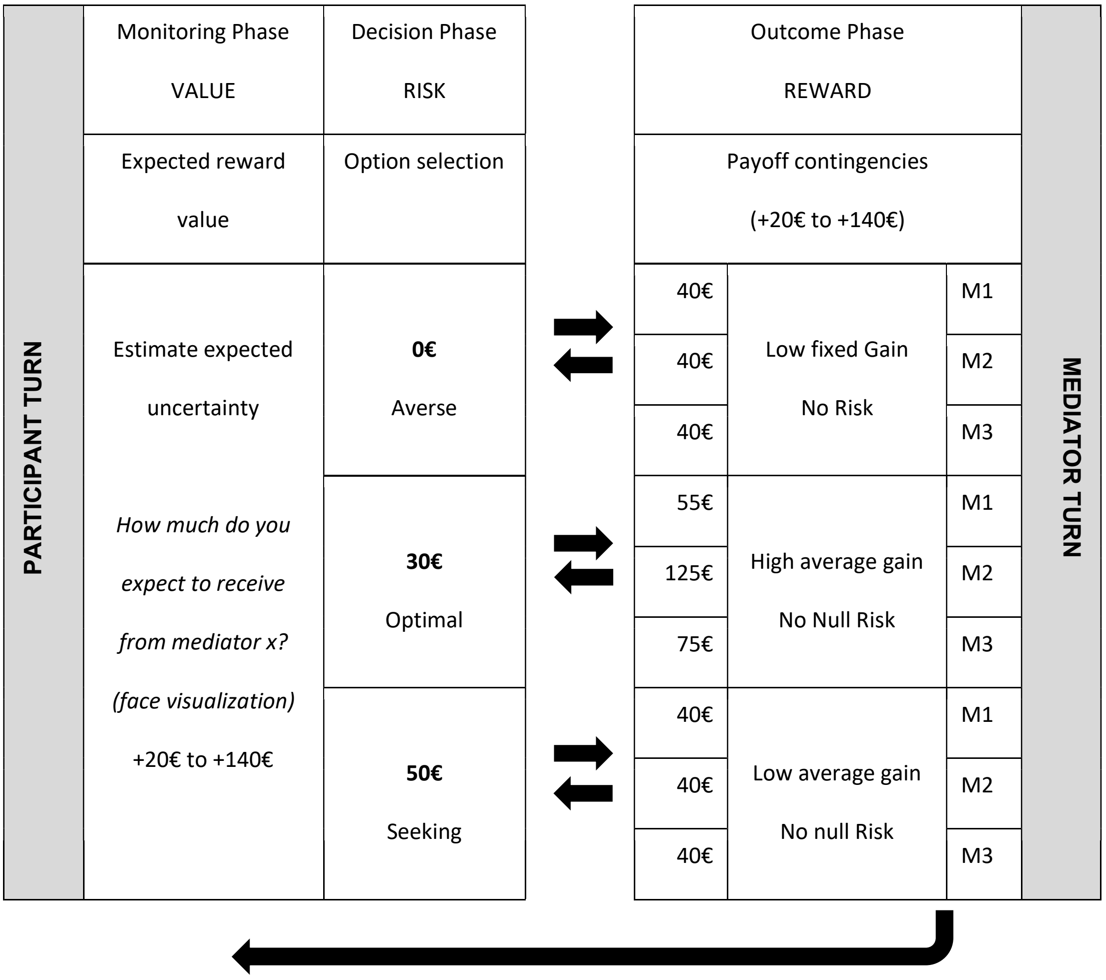

Figure S1. (A) Example of *economic experimental design* considering a run sequence in trust-trustee interaction. Mediator 1 has a low range for reward (trust investment is quite reciprocated, seeming a social norm violation). Mediator 2 has an extreme range, reinforcing optimal decision. Mediator 3 has a moderate range, in the middle of M1 and M2 profile (trust investment is reciprocated in a moderate way, even so seeming a social norm violation). Outcome reward also differed according to participant option (0, 30 or 50 euros) for all mediators.1. For *"0" option (no risk investment) was received a known* low fixed gain (*40 euros;)* 2. For *"50 euros" option (risk investment) was offered a* low average gain (same mean reward, *(40 euros)* that can vary from 20 to 60 euros; 3. For *"30 euros" option (adjusted risk) was earned a* high average gain - low, extreme and moderate reward-: Mediator 1 [35-75]; Mediator 2 [100-140]; Mediator 3 [55-95]. All of them have the same interval (40).

♦ *Health Trust Game and payoff contingencies*

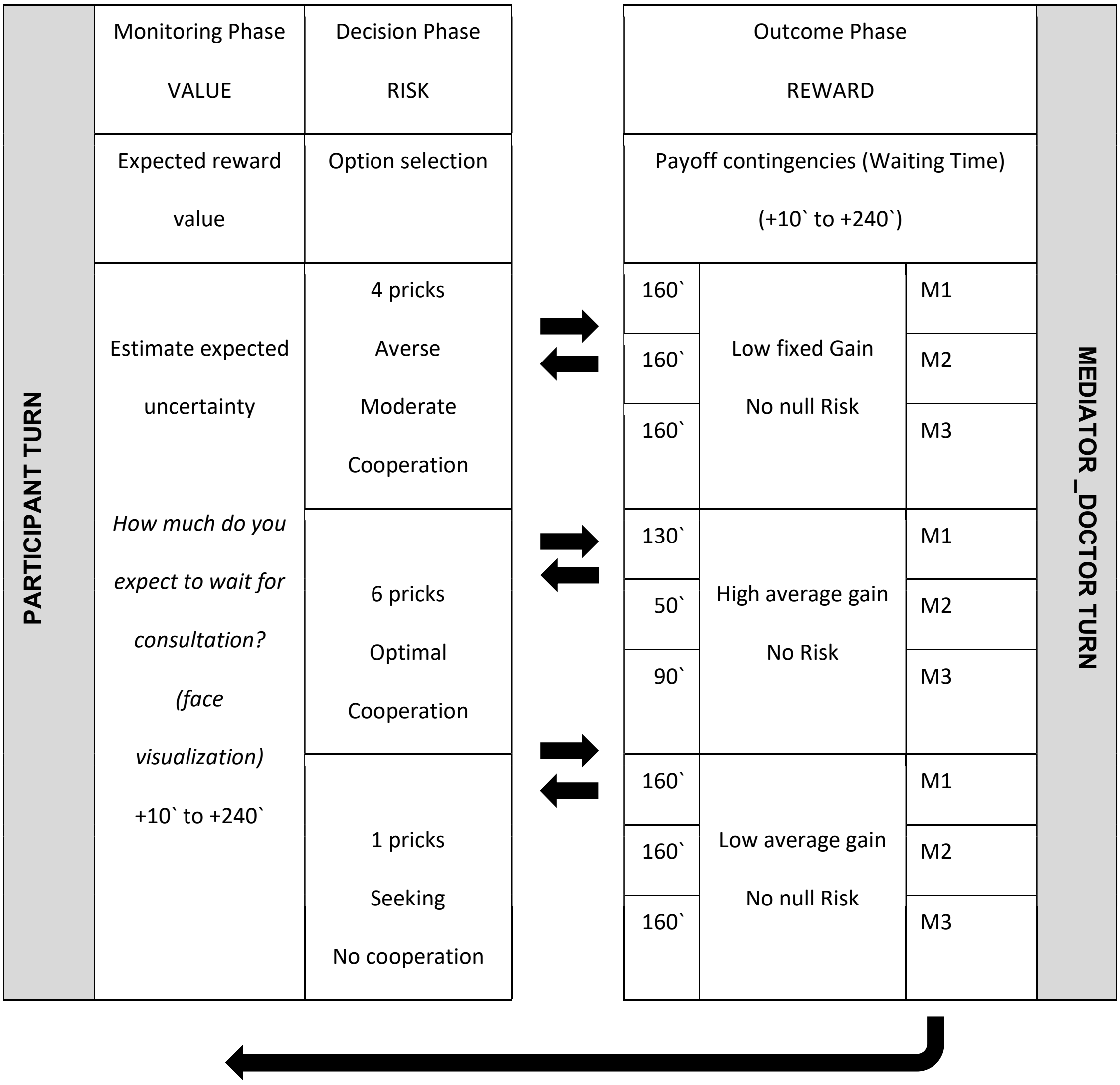

Figure S2 Example of *health experimental design* considering a run sequence in doctor-patient interaction. Mediator 1 has a low range for reward (patient collaboration is quite reciprocated, seeming a social norm violation). Mediator 2 has an extreme range, reinforcing optimal decision fulfilling the pre-established rule. Mediator 3 has a moderate range, in the middle of M1 and M2 profile (patient collaboration is reciprocated in a moderate way, even so seeming a social norm violation). Outcome reward also differed according to participant option (1,4 or 6 pricks) for all mediators.1. For *"4" option (moderate cooperation) was received a known* low fixed gain (*160`)* 2. For *"1" option (no cooperation) was offered a* low average gain (same mean reward, *160`)* it can vary from 120 to 160 minutes. 3. For *"6" option (highest cooperation) was earned a* high average gain - low, extreme and moderate – Mediator 1 [90-170]; Mediator 2 [10-90]; Mediator 3 [50-130]. All of them have the same interval [80].
